# Magic Peptide: Unique Properties of the LRR11 Peptide in the Activation of Leukotriene Synthesis in Human Neutrophils

**DOI:** 10.3390/ijms22052671

**Published:** 2021-03-06

**Authors:** Galina M. Viryasova, Ekaterina A. Golenkina, Tibor Hianik, Nataliya V. Soshnikova, Nina G. Dolinnaya, Tatjana V. Gaponova, Yulia M. Romanova, Galina F. Sud’ina

**Affiliations:** 1 Belozersky Institute of Physico-Chemical Biology, Lomonosov Moscow State University, 119234 Moscow, Russia; gali-inimitable@yandex.ru (G.M.V.); golyesha@mail.ru (E.A.G.); 2 Department of Nuclear Physics and Biophysics, Comenius University, Mlynska dolina F1, 842 48 Bratislava, Slovakia; tibor.hianik@fmph.uniba.sk; 3 Institute of Gene Biology, Department of Eukaryotic Transcription Factors, Russian Academy of Sciences, Vavilov Str. 34/5, 119334 Moscow, Russia; nsoshnikova@genebiology.ru; 4 Department of Chemistry, Lomonosov Moscow State University, 119234 Moscow, Russia; dolinnaya@hotmail.com; 5 National Research Center for Hematology, Russia Federation Ministry of Public Health, 125167 Moscow, Russia; gaponova.tatj@yandex.ru; 6 Gamaleya National Research Centre of Epidemiology and Microbiology, 123098 Moscow, Russia; genes2007@yandex.ru

**Keywords:** neutrophil, leukotriene, oligodeoxyribonucleotides, LRR11, acoustic method, Langmuir isotherm

## Abstract

Neutrophil-mediated innate host defense mechanisms include pathogen elimination through bacterial phagocytosis, which activates the 5-lipoxygenase (5-LOX) product synthesis. Here, we studied the effect of synthetic oligodeoxyribonucleotides (ODNs), which mimic the receptor-recognized sites of bacterial (CpG-ODNs) and genomic (G-rich ODNs) DNAs released from the inflammatory area, on the neutrophil functions after cell stimulation with *Salmonella typhimurium*. A possible mechanism for ODN recognition by Toll-like receptor 9 (TLR9) and RAGE receptor has been proposed. We found for the first time that the combination of the magic peptide LRR11 from the leucine-rich repeat (LRR) of TLR9 with the CpG-ODNs modulates the uptake and signaling from ODNs, in particular, dramatically stimulates 5-LOX pathway. Using thickness shear mode acoustic method, we confirmed the specific binding of CpG-ODNs, but not G-rich ODN, to LRR11. The RAGE receptor has been shown to play an important role in promoting ODN uptake. Thus, FPS-ZM1, a high-affinity RAGE inhibitor, suppresses the synthesis of 5-LOX products and reduces the uptake of ODNs by neutrophils; the inhibitor effect being abolished by the addition of LRR11. The results obtained revealed that the studied peptide-ODN complexes possess high biological activity and can be promising for the development of effective vaccine adjuvants and antimicrobial therapeutics.

## 1. Introduction

Human neutrophils (or polymorphonuclear leukocytes, PMNLs) play an essential role in the initiation and resolution of the inflammatory response to bacterial and viral infections. They eliminate pathogens through phagocytosis, which activates the 5-lipoxygenase (5-LOX)-mediated pathway leading to the synthesis of leukotrienes. Recently, it was found that the leukotriene synthesis in human neutrophils is regulated by genome G-rich DNA fragments and bacterial DNA degradation products—oligodeoxyribonucleotides (ODNs) containing unmethylated CpG motifs (CpG-ODNs) that are released from the inflammatory region [1,2,3]. Using synthetic oligonucleotides that mimic these DNA fragments, it was shown that G-rich ODNs capable of folding into non-B form G-quadruplex structures activated bacterial phagocytosis and the leukotriene synthesis in human neutrophils, both the secondary structure of ODNs and their ability to penetrate into cells being important for the induction of immune responses [2].

In neutrophils, bacteria and pathogen-associated molecular patterns (PAMPs) that are expressed by microorganisms are recognized by Toll-like receptors (TLRs) [4,5,6]. In particular, the mechanisms of immunoregulatory activity of CpG-containing bacterial or synthetic DNA fragments include their interaction with TLR9 receptors, known as DNA sensors [7]. Therefore, synthetic peptides that mimics DNA recognizing regions of the receptor can be effective modulators of neutrophil cell responses. The crystal structure of TLR9 was analyzed [7] and it was predicted that leucine-rich repeats (LRRs) of this receptor are involved in DNA recognition. It was then experimentally shown that the LRR11 peptide derived from the TLR9 sequence can bind to the CpG-ODNs [8], which leads to an attenuating of TLR9 signaling and a decrease in the oligonucleotide internalization by a mouse-derived monocyte/macrophage cell line RAW 264.7 [9].

The LRR11 peptide (QLRKLNLSFNYQKRVSFAHLSLAPSFGSLV) consists of 30 amino acids with a large number of hydrophobic residues (11) and 4 positively charged residues (Arg or Lys); molecular weight 3421 Da. In addition to the fact that this peptide, due to its structural identity with the TLR9 regions, could potentially be its inverse agonist, if not antagonist, it is possible that LRR11 has its own activity. The structure of this peptide is similar to that of cationic host defense peptides (HDPs), which usually contain a large number of positively charged and hydrophobic residues [10]. Direct antimicrobial functions of HDPs leading to membrane permeability and destabilization have been discussed over the years [11], but their microbicidal functions are substantially suppressed by divalent cations Mg^2+^, Ca^2+^, present in physiological solutions [12]. It is now generally accepted that HDPs modulate the functions of immune cells under physiological conditions and work as an innate defense regulator [13,14]. HDP dysregulation, for example, abnormally high level of the cationic antimicrobial peptide LL-37, leads to psoriasis [15]. The absence of HDPs results in severe periodontal disease [16], i.e., these peptides are multifaceted mediators of the immune system. HDPs influence the mobilization of intracellular calcium ions in neutrophils [17,18].

It is assumed that the LRR11 peptide can specifically interfere with the effects of ODNs in human neutrophils. Interestingly, neutrophils express functional TLR9 on the cell surface as well as endosomally, while only endosomal TLR9 is present in other cells. Landau et al. showed that surface TLR9 may be involved in neutrophil activation under the action of CpG-containing ODNs [19]. 

In this work, we first examined the effect of the LRR11 peptide, which is identical to TLR9 subdomain, by itself and in combination with synthetic ODNs of different classes on the leukotriene synthesis, the level of cytoplasmic Ca^2+^ concentration, and the synthesis of reactive oxygen species in neutrophils. The role of other neutrophil receptors, in particular RAGE, in the synthesis of 5-LOX products was also evaluated. The main goal of this study is to determine the factors that regulate the efficiency of signaling from synthetic oligonucleotides.

## 2. Results

To characterize the combined effect of oligonucleotide and LRR11 peptide on the leukotriene synthesis in human neutrophils, we used oligonucleotides, known as activators (CpG-ODNs) or inhibitors (G-rich ODNs) of TLR9 signaling. They included synthetic CpG-ODNs of different classes, A, B and C, which differ in length, primary and secondary structures, as well as the presence of modified fragments with phosphorothioate internucleotide bonds, and G-rich ODNs containing human telomeric tandem repeats. It has been shown that oligoguanosine tracts, especially those containing phosphorothioate internucleotide bonds instead of natural phosphodiester ones, attached to the ends of synthetic ODNs, significantly increase ODN cellular uptake and improve their immunomodulatory activity [20]. CpG-ODNs with a fully modified phosphorothioate backbone are currently used for most in vivo and in vitro studies, since this modification protects the ODNs from nuclease attack [21].

### 2.1. Effect of ODNs and LRR11 Peptide on 5-LOX Metabolite Synthesis

Human neutrophils synthesize 5-LOX metabolites (leukotriene B4 (LTB4) and its isomers, ω-OH-LTB4 and 5-HETE), during phagocytosis of opsonized zymosan or *S. typhimurium* bacteria [22,23]. In the absence of bacteria or other targets for phagocytosis, treatment of neutrophils with ODNs listed in Table 1 or with LRR11, produce less than 1 ng of LTB4, its isomers, ω-OH-LTB4, and 5-HETE per 10^7^ PMNLs. The potential of the LRR11 peptide and ODNs to regulate the synthesis of products catalyzed by 5-LOX was assessed after cell incubation with ODNs (+/−LRR11) followed by the addition of opsonized *S. typhimurium* bacteria (OS). To test whether neutrophil incubation with ODNs, LRR11 and bacteria affects cell viability, we assessed phosphatidylserine externalization and the integrity of neutrophil membranes after 20 min with ODNs (+/−LRR11) followed by 15 min with OS at 37 °C. The data obtained by flow cytometry using double Alexa Fluor-conjugated Annexin V/propidium iodide staining indicate that in our experiment the percentage of viable cells exceeds 96% (Figure S1).

**Figure 1:**
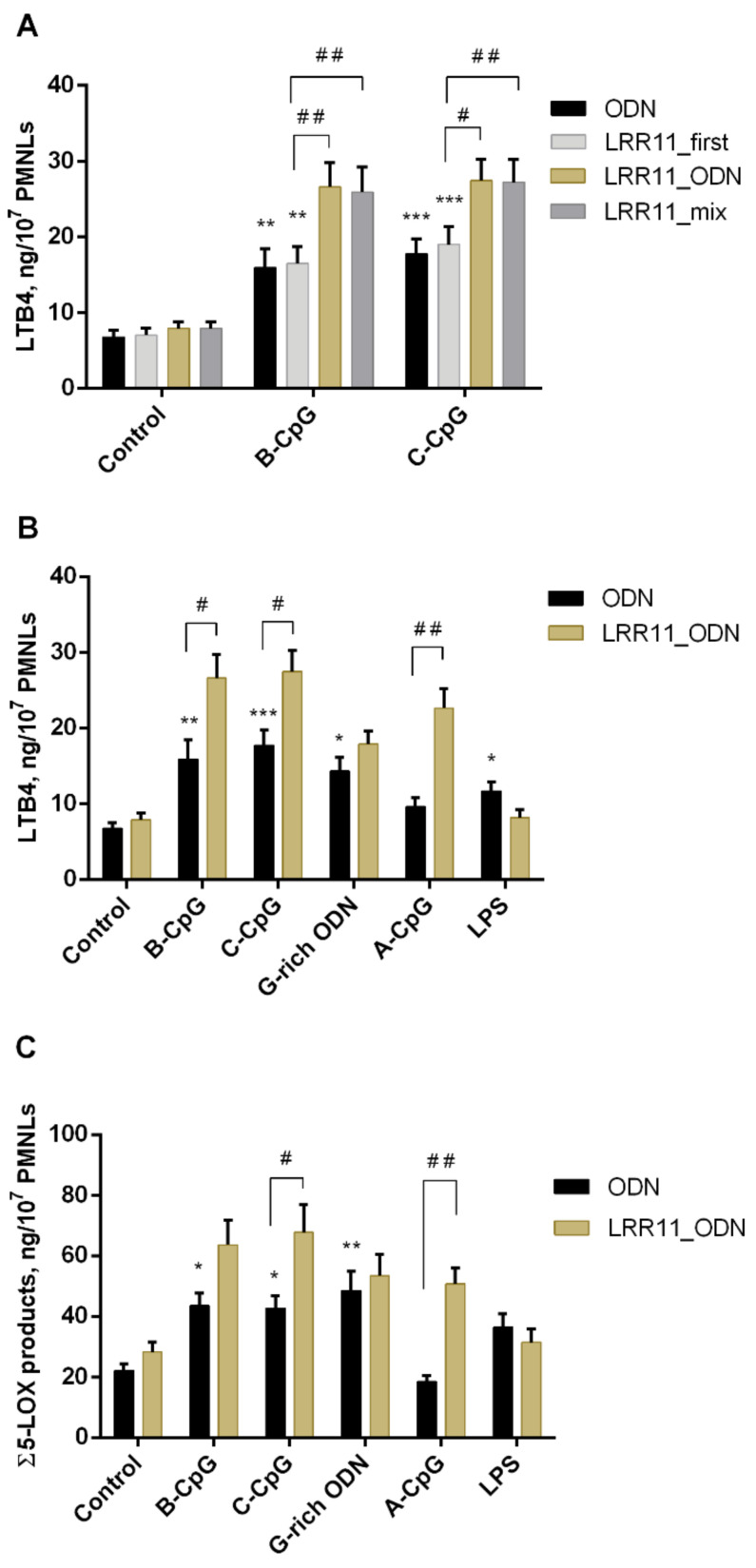
Effects of LRR11 and ODNs on the formation of LTB4 (**A**,**B**) and the sum of 5-LOX metabolites (ω-OH-LTB4, LTB4 and its isomers, and 5-HETE) (**C**) in human PMNLs. PMNLs (2 × 10^7^) were incubated for 15 min at 37 °C without (black bars) or with 1 μM LRR11 (A, LRR11_first); then ODNs and LRR11 or LPS were added; various ways of adding ODN and LRR11 peptide are indicated. In control samples, Hanks’ solution was added instead of ODNs. After 20 min, 0.5 × 10^9^ *S. typhimurium* bacterial cells were added for a further 15 min. The 5-LOX products were determined using HPLC. Values indicate mean ± SEM of five independent experiments performed in duplicate. * *p* < 0.05, ** *p* < 0.01, *** *p* < 0.005, compared with the control. # *p* < 0.05, ## *p* < 0.01 for pairs of data compared as indicated by two-way ANOVA followed by Tukey’s multiple comparison test.

**Figure 2:**
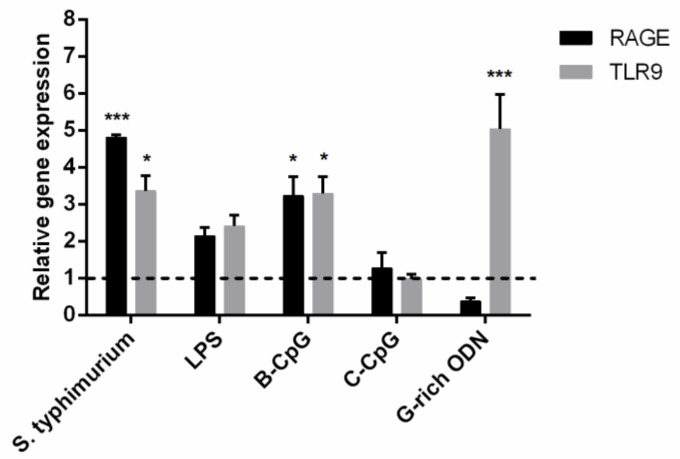
Relative transcription levels of genes encoding RAGE and TLR9 proteins in human neutrophils (2 × 10^6^ /mL) after 1h incubation at 37 °C with the addition of 0.5 × 10^8^ *S. typhimurium* bacteria, 1 µg/mL LPS or 1 µM ODNs. Values measured by qRT-PCR are normalized to vehicle (HBSS/HEPES) control (dotted line). Data are means ± SEM from three independent experiments. * *p* < 0.05, *** *p* < 0.005, compared with the control. Two-way RM ANOVA followed by Dunnett’s multiple comparison test was used.

First, we verified whether the effect of ODNs and LRR11 on the synthesis of 5-LOX metabolites depends on the way the LRR11 peptide is added to neutrophils. We added LRR11 (1) 15min before ODN (denoted as LRR11_first in Figure 1A); (2) we added ODN and LRR11 simultaneously but independently (denoted as LRR11_ODN), or (3) as pre-mixed solution: 1-h pre-mixing of LRR11 with each of the ODNs (denoted as LRR11_mix) (Figure 1A). 

It turned out that only pathways (2) and (3) provide a significant effect on LTB4 accumulation, the magnitude of which practically does not depend on whether the ODN and the peptide were added to the neutrophil cells simultaneously, but independently or were pre-mixed. Under the optimal conditions found, the combined action of ODN and LRR11, added simultaneously to neutrophil cells, on the production of LTB4 (Figure 1B) and the sum of 5-LOX metabolites (Figure 1C) was characterized. As can be seen, the LRR11 peptide does not significantly change the effects of G-rich ODN and lipopolysaccharides (LPS), but obviously enhances the effects of CpG-ODNs.

In other words, the addition of LRR11 to CpG-ODNs of all classes stimulated the formation of the 5-LOX products (Figure 1). However, the effect of various types of CpG-ODNs on the synthesis of 5-LOX metabolites was different. Indeed, A-class CpG-ODN 2216 decreased the synthesis of leukotrienes [3]. A-class CpG-ODN 2336 used in this study did not significantly affect 5-LOX activity, but B- and C-class CpG-ODNs increased leukotriene synthesis, as did G-rich ODN. CpG-ODNs are known to interact with TLR9, and it can be assumed that inhibition of this interaction by the LRR11 peptide derived from TLR9 receptor increases 5-LOX product synthesis. Chloroquine, a small molecule inhibitor of TLR9 signaling, is known to inhibit CpG-ODN–induced cell activation [25]. It has been shown that the inhibition of 5-LOX product formation by ODN 2216 (A-class CpG-ODN) is abrogated by chloroquine [3], but in our experiments, the effects of B- and C-class CpG-ODNs were not sensitive to chloroquine (Table S1). We hypothesize that some receptors on the cell surface have a multivalent capacity to bind a broad spectrum of ligands, including ODNs, and that the stimulatory action of LRR11 on CpG-ODNs may be related to cooperation with other receptors on the surface of neutrophils.

### 2.2. RAGE Inhibitor FPS-ZM1 Attenuates the Potency of ODNs to Increase 5-LOX Products Synthesis in PMNLs, But LRR11 Peptide Abolishes the Effects of FPS-ZM1

Together with TLR receptors, RAGE is part of a family of receptors that recognize pathogen-associated molecules, such as bacterial DNA. RAGE binds to extracellular DNA, facilitating its recognition by TLR9 [26]. The basic level of RAGE expression is known to be very low; however, when cells are exposed to pathogens, the RAGE expression is increased [27]. In response to pathogen, RAGE migrates to cell membranes [28]. 

To begin with, we showed that *S. typhimurium* bacteria, *S. typhimurium* LPS, and CpG-ODNs increase the expression of RAGE receptors on PMNLs (Figure 2). Therefore, we have proved that in our experimental model of neutrophil interaction with bacteria, *S. typhimurium* bacteria significantly increase the expression of these two genes and can mask the action of other agents.

**Figure 3:**
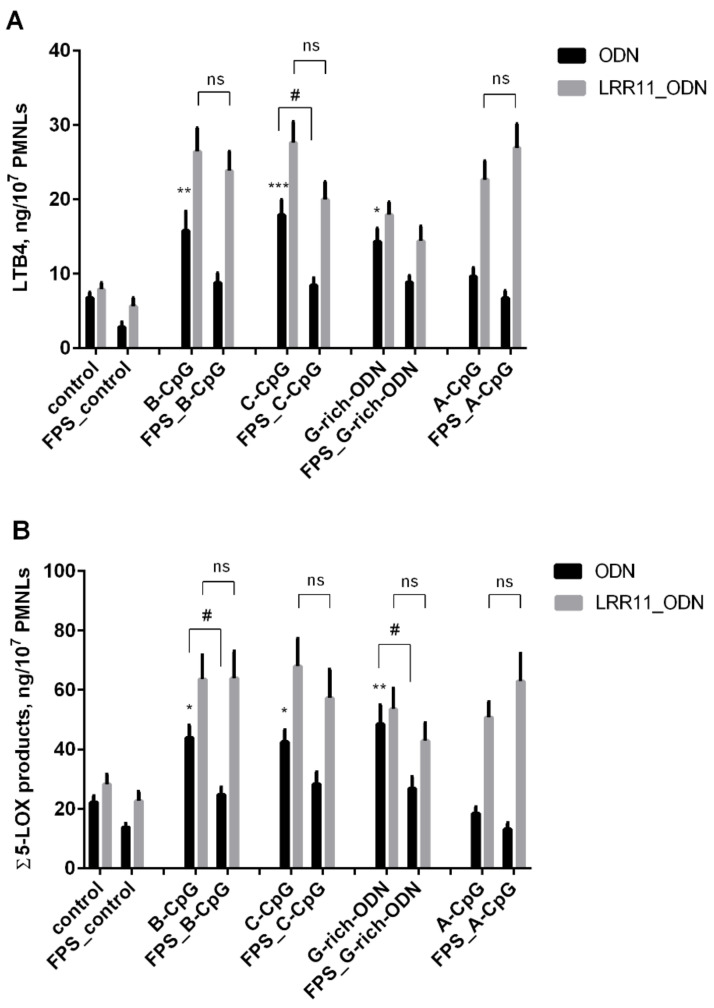
Synthesis of the 5-LOX metabolites in PMNLs exposed to opsonized *S. typhimurium* bacteria in the presence of the RAGE antagonist FPS-ZM1. PMNLs (2 × 10^7^) were incubated for 15 min at 37 °C without additives or with 0.15 µM FPS-ZM1, for the next 20 min the mixture was incubated with 1 µM ODNs without (black bars) or with 1 µM LRR11 (gray bars) before adding 5 × 10^8^ *S. typhimurium* for 15 min. In control samples, Hanks’ solution was added instead of ODNs. The 5-LOX products were determined using HPLC. Graphical data are presented for LTB4 (**A**) or the sum of LTB4 and its isomers, ω-OH-LTB4 and 5-HETE, designed as Σ5LOX products (**B**). Values indicate mean ± SEM for four independent experiments. * *p* < 0.05, ** *p* < 0.01, *** *p* < 0.005 versus the corresponding control. # *p* < 0.05, ns–no significant difference, for pairs of data compared as indicated by one-way ANOVA followed by Tukey’s multiple comparison tests.

We then investigated the effect of ODNs on 5-LOX product synthesis in the presence of FPS-ZM1, a high-affinity RAGE-specific inhibitor [29]. FPS-ZM1 at 150 nM concentration was shown to strongly reduce the effects of ODNs on leukotriene synthesis, but its action is nullified in the presence of LRR11 peptide (Figure 3). FPS-ZM1 also inhibited the 5-LOX product formation stimulated by bacterial phagocytosis (Figure 3) but did not affect the leukotriene synthesis induced by the calcium ionophore A23187 (Table 2).

Taken together, our results suggest that the LRR11 peptide reduces the interaction of the ODN with the TLR9 receptor, “turns off” RAGE inhibition, and stimulates 5-LOX product synthesis in the same way as for neutrophil treatment with CpG-ODNs or G-rich ODN. Thus, more efficient ODN signaling in the presence of LRR11 peptide may be related to cooperation between several receptors on the neutrophil surface, TLR9 and RAGE, which are capable of recognizing DNA fragments.

### 2.3. The Ability of the LRR11 Peptide to Form a Complex with ODNs of Different Sequences

To characterize the interaction of the studied synthetic ODNs with the LRR11 peptide, we applied the thickness shear mode (TSM) acoustic biosensor method, which is based on the investigation of ultrasonic waves in piezoelectric materials such as AT-cut quartz. This approach involved immobilizing a 5′-biotinylated ODN on the surface of TSM transducer covered by NeutrAvidin and then adding the LRR11 peptide. An unrelated peptide (URP) with a random primary structure (LQTLDLRDNALTTIHFIPSIPD) was used as a negative control [9]. The measurement procedure was divided into several steps, in which each immobilization stage was followed by washing with an appropriate buffer solution; when peptide solutions at different concentrations (from 1 to 100 nM) were added to the surface-modified TSM transducer, a wash with Hanks buffer was used before increasing the peptide concentration.

**Figure 4:**
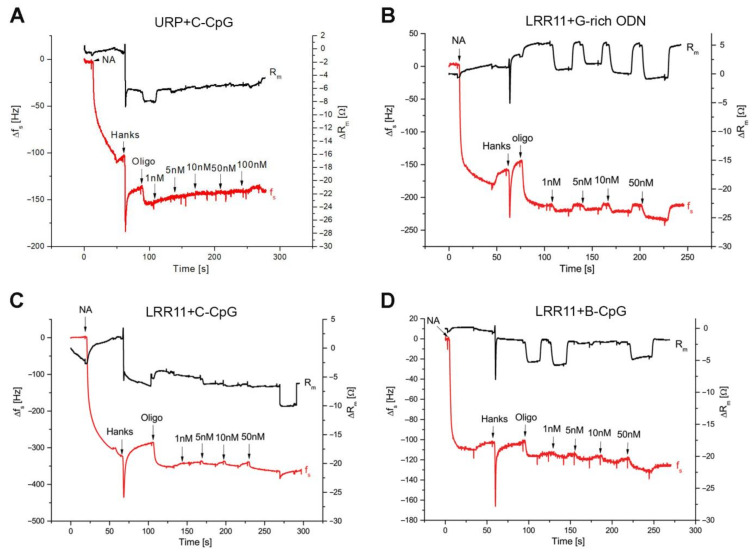
The representative kinetic curves of the resonance frequency (∆*f*_s_, red) and the motional resistance (∆*R*_m_, black) changes that occurred after adding peptide: URP (**A**) or LRR11(**B**–**D**) at different concentrations (nM), to the surface of a TSM transducer modified by NeutrAvidine with attached 5′-biotinylated ODNs. Peptide additions are indicated by arrows. Hanks buffer solution was used to replace media after NeutrAvidine immobilization on a TSM transducer.

The kinetic curves of the changes of resonance frequency (∆*f*_s_), and the motional resistance (∆*R*_m_), which followed various surface modifications, are presented in Figure 4. The addition of NeutrAvidin resulted in a sharp decrease of resonance frequency by approximately 195.65 Hz and in a slight decrease of motional resistance of about 1.1 Ohm. A decrease of the *f*_s_ value suggests the chemisorption of NeutrAvidin on the gold layer of the crystal, while a decrease of the *R*_m_ is an evidence of a negligible viscosity contribution [30]. ODN added to the sensor resulted in a decrease of the resonance frequency caused by the strong binding of the biotin-modified ODN to NeutrAvidin. The motional resistance increase that accompanies oligonucleotide binding suggests a viscosity contribution of the ODN layer due to decreased molecular slip between the ODN chains and the buffer solution [31]. We have proven that there is no unspecific biding between NeutrAvidin and the LRR11 peptide (or URP) in the absence of ODNs.

Typical kinetic changes in f_s_ and *R*_m_ values after the addition of control URP peptide at the surface of a TSM transducer with immobilized C-CpG are shown in the Figure 4A. It can be seen that no frequency reduction was occurred even after the addition of 100 nM URP. Similar results were obtained also for B-CpG and G-rich ODN, which confirm the absence of interactions between the URP and ODNs under consideration. In further experiments, LRR11 was applied to the ODN-modified sensor surface instead of URP (Figure 4B–D). Importantly, the G-rich ODN does not interact with the LRR11 peptide at any concentration. For the G-rich ODN, a slight decrease of the resonance frequency by 5–10 Hz was abolished after washing with buffer solution, as shown in Figure 4B. In contrast, oligonucleotides B-CpG and C-CpG demonstrate specific interactions with LRR11. Despite not significant or small changes of ∆f_s_ was observed for low concentrations of LRR11 (1, 5, 10 nM), an increase in the peptide concentration to 50 nM led to a decrease of the resonance frequency (after washing) by 17 Hz for C-CpG (Figure 4C) and by 12 Hz for B-CpG (Figure 4D).

**Figure 5:**
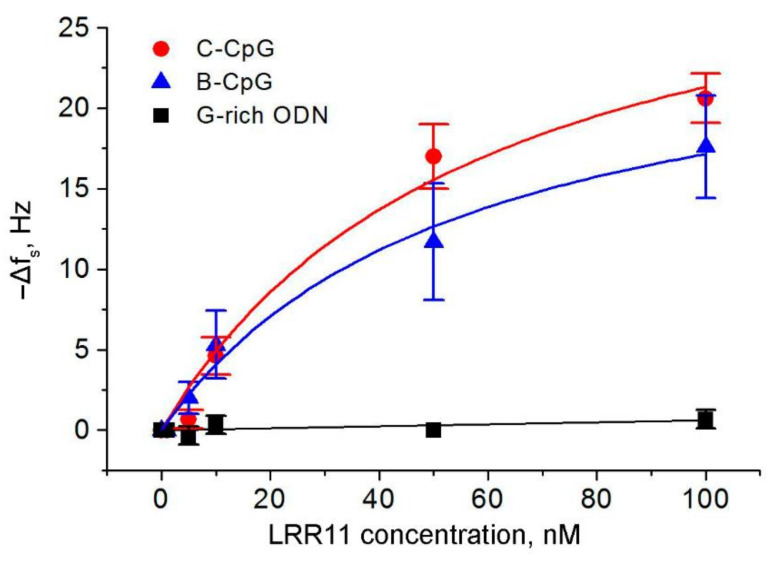
Plot of changes in the resonance frequency, ∆f_s_, as a function of the LRR11 peptide concentration for an acoustic biosensor with immobilized 5′-biotinylated ODNs under investigation. The data represent mean ± SEM obtained from three independent experiments. The curves are fitted according to Equation (1) obtained with the OriginPro 7.5 (OriginLab Corporation, MA, USA).

The dependence of ∆*f*_s_ change as a function of LRR11 concentration is presented in Figure 5. The maximum change was about 20.6 ± 1.53 Hz for the C-CpG ODN after the addition of 100 nM LRR11, while no significant change of resonant frequency was observed when the peptide was added to the G-rich ODN, as already mentioned. The data for 100 nM LRR11 were obtained in separate experiments in which the peptide was added at this concentration to the surface of TSM transducer after its washing with Hanks buffer.

Based on Langmuir isotherm Equation (1),

(1) $$\Delta f_{c}\left(c\right)={\left(\Delta f_{s}\right)}_{\max}\left[c/\left(K_{D}+c\right)\right]$$

where Δ*f*_s_(c) is the frequency change at a certain peptide concentration under equilibrium conditions, (Δf_s_)_max_ is the maximum frequency change, c is the peptide concentration, and *K*_D_ is the equilibrium constant of dissociation, we estimated the (Δ*f*_s_)_max_ and *K*_D_ values for binding of the LRR11 peptide to C-CpG and B-CpG (Table 3).

For G-rich ODN, the Langmuir isotherm cannot be plotted due to the absence of significant changes in the resonance frequency. The *K_D_* values for C-CpG and B-CpG did not differ significantly. This means that, under equilibrium conditions, the stability of the LRR11 complexes with both oligonucleotides is similar, despite the richer secondary structure of C-CpG. This ODN forms a DNA hairpin, where the Gibbs free energy, ΔG, is −3.4 kJ/mol (data obtained using the fold program (https://rna.urmc.rochester.edu/RNAstructureWeb/, accessed on 15 December 2020), while no folding is observed for B-CpG. However, this hairpin structure probably does not form special binding site for LRR11. The *K*_D_ values are relatively low and close to those for specific binding of some proteins to DNA aptamers (1–100 nM) [32,33,34]. 

Using Sauerbrey’s Equation (2),

(2) $$\Delta f=-\frac{2f_{0}^{2}\Delta m}{A\sqrt{\mu_{q}\rho_{q}\;}}$$

where *f*_0_ is the fundamental resonance frequency (in our case 8 MHz), Δ*m* is the change in mass (in grams) of the adsorbed layer on the crystal surface, A is the effective crystal area (in our case 0.2 cm^2^), μ*_q_* = 2.947 × 10^11^ g × cm^−1^s^−2^ is the shear modulus of elasticity, and ρ*_q_* = 2.648 g × cm^−3^ is the crystal density [35], surface concentrations of NeutrAvidin, ODN and LRR11 peptide were estimated based on changes of resonance frequency (Figure 4). According to Equation (2), the surface density of molecules, σ (in molecules/cm^2^), can be calculated as σ = Δ*m*N_A_/(*A ×* Mw), where N_A_ is Avogadro’s number (=6.023 × 10^23^ mol^−1^), and Mw represents the molecular weight of the corresponding compound (NeutrAvidin: 65 kDa, 5′-biotinylated C-CpG: 7115.4 Da, and LRR11: 3421 Da). Based on this approach, we obtained the following surface concentration values: 1.94 × 10^13^ cm^−2^ for NeutrAvidin, 3.37 × 10^13^ cm^−2^ for C-CpG and 2.4 × 10^13^ cm^−2^ for LRR11. The NeutrAvidin has four binding sites, two of which are available for biotinylated ODN binding. This is due to the fact that the other two sites are inaccessible due to NeutrAvidin chemisorption on the TSM surface. Thus, the number of available binding sites for the ODN is 3.88 × 10^13^ cm^−2^. This is comparable to the surface concentration of C-CpG ODN. Therefore, the surface of the TSM transducer is practically completely saturated by C-CpG. Estimation of the surface concentration of LRR11 evidences that at 50 nM concentration of this peptide, it occupies almost 70.6% of the ODN molecules.

### 2.4. The LRR11 Peptide Promotes the Uptake of Synthetic ODNs by Neutrophils

It is known that synthetic oligonucleotides of various primary and secondary structures can exhibit unequal ability to bind to the outer cell membrane and internalize into the intracellular space. Obviously, these parameters will vary for different types of cells. We used fluorescein amidite (FAM)-labeled oligonucleotides for a comparative analysis of LRR11 peptide effects on the interaction of ODNs with neutrophils.

**Figure 6:**
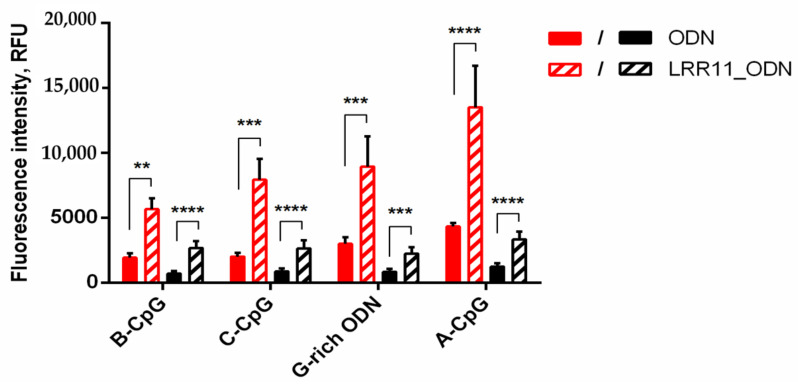
The LRR11 peptide affects the binding and uptake of ODNs by neutrophilic granulocytes. PMNLs (1 × 10^6^/mL) in HBSS/HEPES were incubated for 20 min at 37 °C in 5% CO_2_ with 1 µM FAM-labeled ODN, supplemented or not with 1 µM LRR11 peptide. Both the total ODN accumulation (red) and its intracellular constituent (black) were analyzed by flow cytometry. Surface fluorescence was quenched by trypan blue (TB) solution. Values represent the means ± SEM of FAM fluorescence intensities (RFU, relative fluorescence units) (n = 8). ** *p* < 0.01; *** *p* < 0.005, **** *p* < 0.001 for specified data pairs, as indicated by two-way ANOVA followed by Tukey’s multiple comparison test. Mean fluorescence intensities of unstained PMNLs were 62 ± 12 and 39 ± 5.6 without or after TB quenching, respectively.

It was shown that the joint addition of the LRR11 peptide and ODN to neutrophils causes a 3–4-fold increase in both the binding of all analyzed oligonucleotides to the outer neutrophils’ membrane and their penetration into cells compared to ODN alone (Figure 6).

### 2.5. RAGE Antagonist Inhibits ODN Internalization, But the Addition of LRR11 Peptide Neutralizes This Effect

**Figure 7:**
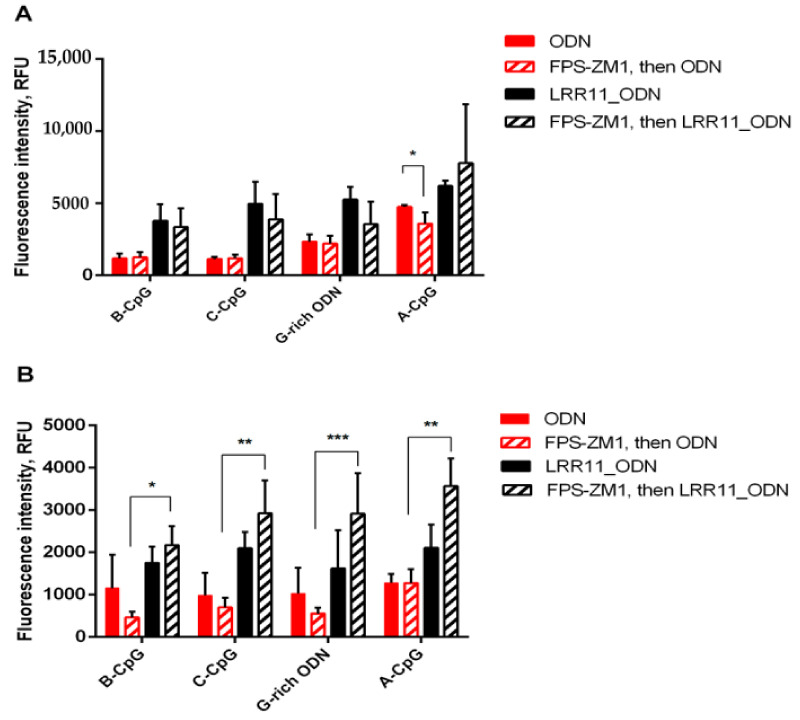
RAGE antagonist effect on oligonucleotide penetration into neutrophil cells. PMNLs (1 × 10^6^/mL) in HBSS/HEPES were incubated for 15 min at 37 °C in 5% CO_2_ with (shaded) or without (solid fill) 0.15 µM FPS-ZM1. Then 1 µM FAM-labeled ODNs, supplemented (black) or not (red) with 1 µM LRR11 peptide, were added for next 20 min. Both total oligonucleotide accumulation (**A**) and its intracellular constituent (**B**) were analyzed by flow cytometry. The surface fluorescence was quenched by trypan blue solution. Values represent the means ± SEM of FAM fluorescence intensities (RFU) (n = 3). * *p* < 0.05; ** *p* <0.001; *** *p* < 0.005 for the pair of data specified, as indicated by two-way ANOVA followed by Tukey’s multiple comparison test. Fluorescence intensities of unstained controls were 62 ± 14 and 49 ± 6 without, or after TB quenching, respectively.

The results obtained confirm that RAGE plays an important role in promoting ODN penetration into cells. Blocking this receptor reduces the effectiveness of ODN internalization. However, the addition of the LRR11 peptide overcomes the inhibitory effect of FPS-ZM1. Moreover, the maximum ODN delivery into cells is observed in the presence of LRR11 and a RAGE antagonist (Figure 7). TLR9 and RAGE cooperate to recognize DNA fragments. It is possible that the RAGE antagonist protects LRR11 from competitive interaction with RAGE and maintains its greater availability for ODNs.

### 2.6. LRR11 Peptide Promotes an Increase in the Production of Reactive Oxygen Species Induced by Oligonucleotides

We have previously shown [2,3] that synthetic oligonucleotides of various types containing phosphorothioate fragments, to one degree or another, induce the generation of reactive oxygen species by neutrophilic granulocytes. Leukotriene synthesis is regulated by multiple mechanisms, and reactive oxygen species not only provide some peroxide tone required to activate 5-LOX, but through the initiation of NADPH-oxidase-dependent superoxide synthesis, lead to a decrease in NOS activity and protect against NO-induced inhibition of 5-LOX [22,36]. 

**Figure 8:**
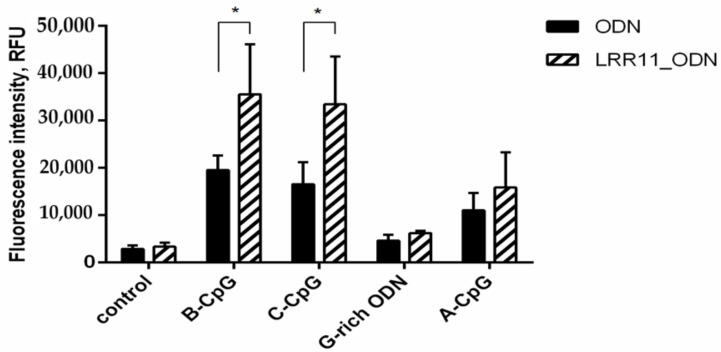
When added simultaneously, LRR11 potentiates the prooxidant effects of oligonucleotides. H_2_DCF-DA-stained PMNLs (1 × 10^6^/mL) were cultured for 1 h at 37 °C in 5% CO_2_ without additives or in the presence of 1 µM ODN, supplemented (shaded) or not (solid fill) with 1 µM LRR11. DCF green fluorescence signals (ex. 485 nm, em. 538 nm) were monitored every 5 min for an hour. Values represent the means ± SEM of DCF fluorescence intensity increments (RFU) in 30 min after stimulus addition (*n* = 3). * *p* < 0.05 for the pairs of data specified, as indicated by two-way ANOVA followed by Tukey’s multiple comparison test.

Notably enhancing the prooxidant potential of the studied ODNs, in particular, CpG-containing ones, the LRR11 peptide itself does not affect the synthesis of reactive oxygen species (Figure 8).

Since leukotriene synthesis in PMNLs during their interaction with bacteria is often correlates with phagocytosis of bacteria or other objects of phagocytosis [22], we estimated the effects of ODNs and LRR11 on the adhesive properties of PMNLs and on phagocytosis of *S. typhimurium*. In fact, all studied ODNs stimulated PMNL adhesion, and only G-rich ODN facilitated the bacteria engulfment; LRR11 did not affect this (Figure S2a,b).

### 2.7. LRR11 Peptide Stimulates an Increase in the Intracellular Concentration of Free Ca^2+^ in Neutrophils

The involvement of calcium ions in the formation of leukotrienes by 5-LOX appears well-established. Numerous studies suggest for this enzyme to be calcium ion dependent. Under the action of the ionophore, 5-lipoxygenase is translocated from the cytosol to the nuclear membrane, where it binds to the activating protein [37]. 

**Figure 9:**
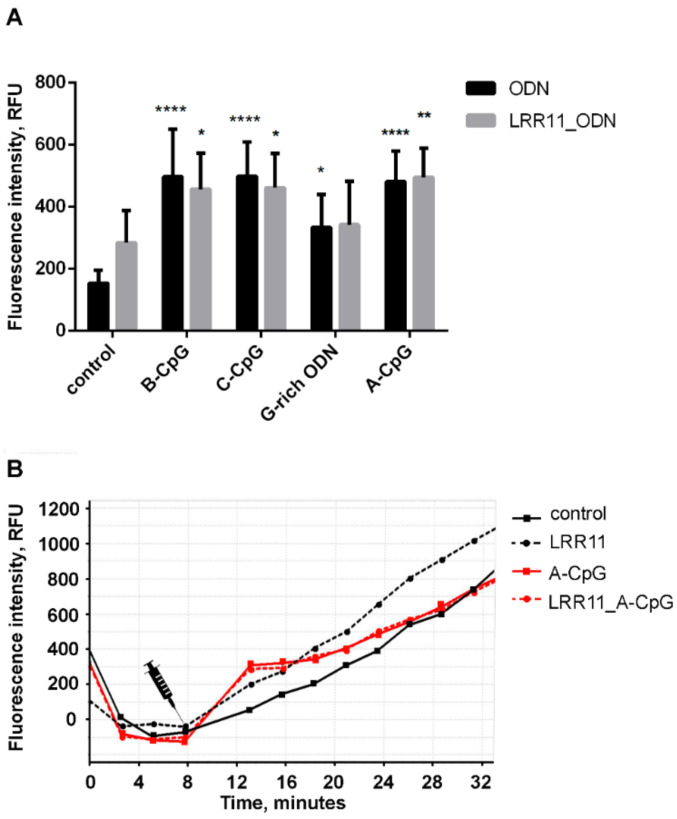
Effects of ODNs and LRR11 on intracellular concentration of free Ca^2+^ in neutrophils. Fluo-3 AM-loaded neutrophils (1 × 10^6^/mL) were cultured in fibrinogen-coated flat bottom plates in HBSS/HEPES medium supplemented or not with 1 µM LRR11, without additives or in the presence of 1 µM ODNs, at 37 °C, 5% CO_2_. Fluo-3 fluorescence signals (ex. 485 nm, em. 538 nm) were monitored with 2-min intervals. (**A**) Values represent the means ± SEM of fluorescence intensity increments in 7 min after the addition of stimulus (*n* = 3). * *p* < 0.05, ** *p* < 0.01, **** *p* < 0.001 compared with the control, as indicated by two-way ANOVA followed by Sidak’s multiple comparison test. (**B**) Representative blank corrected Fluo-3 fluorescence kinetic curves for control (square, black solid line), LRR11 (circle, black dotted line), A-CpG (square, red solid line) and LRR11_A-CpG mix (circle, red dotted line).

All studied oligonucleotides, especially the CpG-ODNs, and the LRR11 peptide potentiates calcium influx. Unlike ODNs, which cause an immediate calcium spurt with its subsequent decline below the reference level, the peptide ensures long-term maintenance of an increased cytoplasmic Ca^2+^ concentration (Figure 9). The effect of LRR11 on the influx of calcium ions was noticeable only in the absence of ODNs.

## 3. Discussion

It is known that the activation of neutrophil TLR receptors is regulated by products of bacterial degradation (unmethylated CpG-containing DNA) and fragments of genomic G-rich DNA released from the inflammatory area. The mechanisms of immunoregulatory activity of CpG-containing bacterial or synthetic ODNs, include their interaction with TLR9 receptors, which are the most studied immune sensors of DNA [7]. On the other hand, synthetic peptides that mimic the DNA recognizing regions of the TLR9 receptor also effectively modulate neutrophil cell responses. 

LRR11, like LL-37, belongs to the group of cationic antimicrobial peptides, which are characterized by high content of hydrophobic residues (~40%) and overall positive charge due to the increased content of Arg and Lys residues [38]. They block many functions of LPS [39,40], interact with intracellular DNA, and mediate DNA uptake and transport in mammalian cells [41]. Therefore, LL-37 peptide forms a complex with telomeric G-quadruplex DNA [42], and promotes the rapid delivery of bacterial CpG-ODNs to cells, and this activity was not associated with the cell-penetrating properties of LL-37, since GL-37 peptide, random analogue that does not possess bactericidal properties, can reproduce the response to LL-37 [43,44]. It is now well-accepted that antimicrobial peptides influence the regulation of inflammation by directly affecting immune cells and inducing the synthesis of immune response mediators [14]. Neutrophil cells are a rich source of cationic antimicrobial peptides [45,46] and positively charged free amino acids [47]. Synthetic cationic peptides modulate the uptake of CpG-ODNs, which enhances the immunostimulatory effects [48,49]. In turn, CpG-ODNs dramatically increase the retention of peptides in early endosomes, thereby enhancing the antitumor immunity of peptides [50].

In our experimental model, leukotrienes and other 5-LOX metabolites, which help regulate leukocyte chemotaxis during inflammation, were produced after neutrophil exposure to *S. typhimurium* bacteria. We showed that, under these conditions, treatment with a combination of CpG-ODN of all classes and the LRR11 peptide noticeably stimulates the formation of 5-LOX products (Figure 1), synergistically amplifying each other’s effects. In contrast, the LRR11 peptide did not significantly alter the effects of G-rich ODN and LPS. These findings are consistent with the literature data that the surface TLR9 receptor may be involved in the activation of neutrophils by CpG-containing ODNs [19]. 

The same patterns were observed when we studied the generation of reactive oxygen species by neutrophilic granulocytes (Figure 8): Among all studied ODNs, only CpG-ODNs in combination with LRR11 enhanced responses.

The ability of the LRR11 peptide to form a complex with ODNs of different sequences and the equilibrium constants of the peptide binding to the ODNs were estimated using an acoustic wave biosensor analysis, which involves immobilization of one of the reactants on the surface of the device. It can be seen that the CpG-ODNs bind with high affinity to the LRR11 peptide, while the G-rich-ODN does not (Figure 4 and Figure 5). These results point to the possibility that CpG-ODNs can interact with TLR9 and be recognized by its LRR11 region. Hence, both individual molecules and specific complexes of CpG–ODN with the LRR11 peptide are involved in the activation of neutrophil responses. We assume that the action of ODNs on neutrophil cells involves several signaling pathways, and the ODN/LRR11 complex has a stronger effect than CpG-ODN or LRR11 peptide alone.

Most likely, CpG-ODN can serve as a linker between various receptor molecules of neutrophils. We hypothesize that some receptors on the cell surface have a multivalent ability to bind a broad spectrum of ligands, including ODNs, and that the stimulatory action of LRR11 on the CpG-ODNs may be related to cooperation with other receptors on the neutrophil surface. It was demonstrated that the extracellular VC1 domain of cell-surface DNA-binding receptor RAGE [26] has a positive charge due to the high content of lysine and arginine residues [51] and can bind to nucleic acids, including phosphorothioate ODNs, through interaction with negatively charged sugar-phosphate backbone in a sequence-independent manner [26]. Therefore, the RAGE ligand CpG-ODN 2216 can form a ternary complex with RAGE and the complement component C3a [52]; another ligand CpG-ODN 2006 after RAGE binding promoted rapid lysosomal degradation of the receptor [53]. Notably, DNA-protein interactions occurring at the cell surface enhance endosomal uptake, involvement of TLR9 receptor, and subsequent NF-κB activation. Inside the cytosol, the RAGE cytoplasmic tail behaves similarly to the domain of the TLR4-TIR complex interacting with the TIRAP and MyD88 adapter molecules, which activate multiple downstream signals [54].

We found that the RAGE receptor is involved in the activation of 5-LOX in neutrophils exposed to *S. typhimurium* bacteria and in ODN-mediated regulation of leukotriene synthesis. RAGE is shown to bind molecules, known as damage-associated molecular patterns (DAMPs), released by cells that die or are under conditions of inflammation. Recently, DAMPs, and in particular the High-Mobility Group Box 1 (HMGB1) protein, have been shown to induce leukotriene production in human monocytes through a RAGE-dependent pathway [55]. It has also been found that LTB4 increases RAGE expression in proliferating human vascular smooth muscle cells and that 5-LOX product synthesis and RAGE expression are closely related [56]. In vascular smooth muscle cells, HMGB1 enhanced RAGE expression along with an increase in LTB4 production [56]. Soluble RAGE inhibited LTB4-dependent neutrophil migration through interaction with the LTB4 receptor, BLT1 [57]. Nothing has been published on the relationship between 5-LOX product synthesis and RAGE regulation in neutrophils; this remains to be seen.

In this context, our experiments with FPS-ZM1, a high affinity RAGE-specific inhibitor [29], are of interest. FPS-ZM1 inhibited the activation of 5-LOX-mediated synthesis induced by ODNs and bacteria, but LRR11 abolished the effects of FPS-ZM1 and restored the level of leukotriene synthesis seen in the presence of the LRR11 peptide and ODN (Figure 3). FPS-ZM1 suppressed ODN internalization, and the most pronounced effect of the LRR11 peptide is its ability to overcome this inhibitory action (Figure 7). The effect on ODN delivery seems to explain the unique properties of the LRR11 peptide in the activation of leukotriene synthesis in human neutrophils.

Based on these findings, we can speculate that more efficient signaling from DNA molecules may be related to cooperation between several receptors on the neutrophil surface, thus fine-tuning cellular responses.

## 4. Materials and Methods

### 4.1. Oligonucleotides and Peptides

All oligodeoxyribonucleotides and their modified analogues with a phosphorothioate backbone listed in Table 1 were synthesized via standard phosphoramidite chemistry and purified (HPLC and PAGE) by DNA-Synthesis (Moscow, Russia). LRR11 and control peptides were synthesized and purified (HPLC) by Syneuro (Moscow, Russia).

### 4.2. Primers, RNA Isolation and cDNA Synthesis

RNA was isolated from human neutrophils using TRI Reagent (Merck KGaA, Darmstadt, Germany) in accordance with the manufacturer’s protocol. cDNA was synthesized using oligo(dT) primers and MMLV reverse transcriptase (Thermo Fisher Scientific, Waltham, MA, USA). Transcription levels of genes encoding RAGE and TLR9 proteins in neutrophils were measured by RT-qPCR (Real-Time PCR System StepOnePlus, Thermo Fisher Scientific, Waltham, MA, USA). Primers used to measure gene expression: for RAGE (For: 5′-CTACCGAGTCCGTGTCTACCA, Rev: 5′-CATCCAAGTGCCAGCTAAGAG) and for TLR9 (For: 5′-CAACAACCTCACTGTGGTGC, Rev: 5′-GAGTGAGCGGAAGAAGATGC). Values were normalized to the house keeping gene RPLP0 (For: 5′-ACTGGAGACAAAGTGGGAGCC, Rev: 5′- CAGACACTGGCAACATTGCG).

### 4.3. Isolation of Neutrophils

Human neutrophils were isolated from freshly taken citrate-anticoagulated peripheral blood from healthy individuals using standard methods [58]. Leukocyte-rich plasma was prepared by sedimentation of erythrocytes with 3% dextran T-500 at room temperature. For purification of granulocytes, centrifugation through Ficoll-Paque (density 1.077 g/mL) was used, followed by hypotonic lysis of the remaining erythrocytes. PMNLs were washed twice in PBS, resuspended at 10^7^ cells/mL (96–97% purity, 98–99% viability) in Dulbecco’s PBS containing 1 mg/mL glucose (no CaCl_2_) and stored at room temperature. All methods were performed in accordance with the relevant instructions and regulations of the Ministry of Health of the Russian Federation and local legislation. 

### 4.4. Preparation of Bacteria

Bacteria (*S. typhimurium* IE 147 strain) obtained from the Collection of Gamaleya National Research Center of Epidemiology and Microbiology (Moscow, Russia) were grown in Luria–Bertani broth to a concentration of 1 × 10^9^ colony-forming units (CFU)/mL. The bacteria were collected by centrifugation at 2000 g and opsonized immediately before the experiment for 30 min in 20% *(v/v)* fresh serum from the same donor whose blood was used to isolate neutrophils. Repeated centrifugation in Dulbecco’s solution was used to wash the bacteria.

### 4.5. Synthesis of 5-LOX Metabolites in Human Neutrophils

The cell suspension (2 × 10^7^ PMNLs in HBSS/HEPES, reaction volume of 6 mL) was cultured for 15 min in an incubator at 37 °C, 5% CO_2_. Then 1.0 µM ODN with or without 1.0 µM LRR11, or 2 µg/mL LPS, was added as indicated. After 20 min, the cells were stimulated with *S. typhimurium* (the ratio of bacteria to PMNLs was approximately 20:1) for the next 15 min [3]. The synthesis of 5-LOX metabolites was stopped by adding an equal volume of methanol at −20 °C with prostaglandin B2 as an internal standard. Samples were stored at −20 °C. The denatured cell suspension was centrifuged, and supernatants designated as water/methanol extracts.

### 4.6. HPLC Analysis of Water/Methanol Extracts

To purify the water/methanol extracts, solid-phase extraction was used on Sep-Pak C18 cartridges (500 mg; Macherey-Nagel, Dueren, Germany), which were conditioned first with methanol, and then with water. Major 5-LOX metabolites were identified by comparing retention times with those of known compounds, as previously described [2]. 

### 4.7. Assessment of Calcium Ion Influx

Intracellular Ca^2+^ ions were detected by staining with Fluo-3, which is known to dramatically increase fluorescence after binding of calcium ions, was used. According to the manufacturer’s protocol, human neutrophils were incubated with 5 μM Fluo-3 AM ester (Thermo Fisher Scientific, Waltham, MA, USA) for 60 min at room temperature, followed by washing in PBS. Cells were then seeded in fibrinogen-coated 96-well plates (1 × 10^6^/mL of HBSS/HEPES) and incubated at 37 °C in 5% CO_2_. Changes in fluorescence intensity upon excitation at 485 nm and emission at 538 nm were monitored at CLARIOstar fluorescence microplate reader for at least 60 min after stimulus injection, followed by data analysis using MARS Data Analysis Software, version 3.30 (both hardware and software are from BMG Labtech, Ortenberg, Germany).

### 4.8. Uptake Assay with FAM-Labeled ODNs

To examine neutrophil binding and uptake, the 5′-FAM-labeled ODNs (DNA-synthesis, Moscow, Russia) were added to a cell suspension (1 × 10^6^/mL of HBSS/HEPES) at a concentration of 0.5 µM for 30 min at 37 °C. After incubation, the samples were placed on ice and topped with ice-cold 0.1% BSA in PBS, followed by centrifugation at 270 g. To evaluate the staining of collected PMNLs with 5′-FAM-labeled ODNs, cells were resuspended and analyzed on the Cytomics FC 500 Flow Cytometry System using CytExpert 1.2 Software (Beckman Coulter, Krefeld, Germany), excitation at 488 nm and emission at 525 nm. To distinguish between internalized and surface-bound ODNs, TB at a working concentration of 1 mg/mL was added to quench FAM surface fluorescence.

### 4.9. Reactive Oxygen Species Formation Assay 

The level of intracellular production of reactive oxygen species was monitored by measuring the green fluorescence of H_2_DCF-DA-labeled oxidized product incorporated into cells according to the manufacturer’s protocol. Briefly, human neutrophils were incubated with 5 μM carboxy-H_2_DCF-DA (Molecular Probe, Eugene, OR, USA) for 60 min at room temperature, followed by rinsing with PBS. The cells were then seeded in fibrinogen-coated 24-well plates (1 × 10^6^/mL of HBSS/HEPES) and incubated according to the experimental protocol for 60 min at 37 °C in 5% CO_2_. Fluorescent cell staining (excitation at 485 nm and emission at 538 nm) was recorded at CLARIOstar fluorescence microplate reader, using MARS Data Analysis Software, version 3.30 (both hardware and software are from BMG Labtech, Ortenberg, Germany).

### 4.10. Detection of LRR11-ODN Interaction by TSM Method

Eight MHz AT-cut piezoelectric quartz crystal transducer was obtained from CH Instruments, Austin, TX, USA. The transducer was covered on both sides with thin layers of gold that served as electrodes (working area 0.2 cm^2^). Prior ODNs immobilization, the crystal was carefully cleaned with basic Piranha solution (29% NH_3_, 30% H_2_O and H_2_O_2_ with volumetric 1:5:1 ratio, respectively) for 25 min. After the thorough cleaning, the crystal was positioned between two silicon O-rings in a flow-through cell [30,33]. The analyte was injected into the cell using a Genie Plus syringe pump (Kent Scientific, Torrington, CT, USA) at a flow rate of 50 μL/min. Each solution was applied until the resonance frequency stabilized. The surface of the transducer was first washed with deionized water, and then 125 μg/mL NeutrAvidin (WWR International GmbH, Vienna, Austria) solution in water was applied. Approximately 15 min were required to stabilize the signal at a constant NeutrAvidin flow rate. This was followed by washing with water to rinse out all non-chemisorbed NeutrAvidin molecules. Immobilization of ODN on the gold surface of the crystals is accomplished using avidin–biotin interactions. A 1 μM 5′-biotinylated ODN solution was injected over the neutravidin-modified surface and subsequently the crystal was washed again with water. The system was then equilibrated using Hanks buffer solution. This buffer was suitable for studying LRR11–ODN binding. Then LRR11 was used at concentrations of 1, 5, 10, 50 and 100 nM (per enzyme monomer) in Hanks buffer. After each addition of LRR11, the sensor surface was washed with Hanks buffer and LRR11 with the higher concentration was added. All binding experiments were carried out at room temperature. Buffer and sample solutions were introduced in a flow-through format. A network analyzer 8712ES (Agilent Technologies, Santa Clara, CA, USA) was used to measure the impedance properties of the sensor and determine *f*_S_ and *R*_m_. 

### 4.11. Statistical Analysis

Data are expressed as mean ± SEM and were analyzed by ANOVA. Data were compared using multiple comparisons tests, where differences were considered significant at *p* < 0.05. The graphs and the statistical data analysis were carried out using GraphPad Prism 8 (https://www.graphpad.com 15 December 2020).

## 5. Conclusions

The ability of synthetic ODNs to influence neutrophil functions has drawn attention to their use in immunotherapy. In this study, we demonstrated that the LRR11 peptide derived from the TLR9 receptor sequence greatly potentiates the delivery of ODNs to neutrophilic cells, gently increases and maintains intracellular calcium ion concentration, and dramatically improves the effect of CpG-ODNs on the synthesis of 5-LOX products in neutrophils. Acoustic wave biosensor analysis confirmed the specific binding of the CpG-ODNs, but not the G-rich ODN, to the LRR11 peptide. According to our data, the stimulating effect of LRR11 on the CpG-ODNs is related to cooperation with the RAGE receptor on the neutrophil surface. The results obtained revealed that the studied peptide-ODN complexes possess high biological activity in our experimental model during phagocytosis of opsonized *S. typhimurium* bacteria by human neutrophils. These findings are of great importance for the development of effective vaccine adjuvants and antimicrobial therapeutics.

## Figures and Tables

**Table 1:** Sequences and designations of oligodeoxyribonucleotides used in this study.

Designation	ODN Primary Structure ^a^	Reference
A-CpG ^b^	5′- ggGGACGACGTCGTGgggggg-3′	[24] ODN 2336
B-CpG ^b^	5′-tcgtcgttttgtcgttttgtcgtt-3′	[24] ODN 2006
C-CpG ^b^	5′-tcgtcgttttcggcgcgcgccg-3′	[24] ODN 2395
G-rich ODN	5′-ggTTAGGGTTAGGGTTAGGGTTAGGGggggg-3′	[2] g_2_-G4-g_5_

^a^ Capital letters denote ODN sequences with natural phosphodiester internucleotide bonds; phosphorothioate-containing nucleotide units are indicated in lowercase letters. ^b^ A-CpG, B-CpG and C-CpG correspond to A-class CpG-ODN 2336, B-class CpG-ODN 2006 and C-class CpG-ODN 2395, respectively.

**Table 2:** Effect of RAGE-specific inhibitor FPS-ZM1 on 5-LOX product formation in PMNLs stimulated by calcium ionophore A23187. Neutrophils (1×10^7^) were incubated for 30 min at 37 °C without or with 150 nM FPS-ZM1, as indicated, then 1 µM A23187 was added for 10 min. Data are presented as mean ± SEM from three independent experiments.

PMNLs+	Σ 5-LOX Metabolites (ng/10^7^ PMNLs)
1 µM A23187	416 ± 37
150 nM FPS-ZM1; +1 µM A23187	438 ± 42

**Table 3:** The (Δ*f*_s_)_max_, equilibrium constants of dissociation, *K*_D_, and association, *K_A_*, (*K_A_* = 1/*K_D_*) of the LRR11-ODN complex, determined using the Langmuir fit (Equation (1)) of the experimental data presented in Figure 5 for the TSM biosensor with immobilized 5′-biotinylated oligonucleotides C-CpG and B-CpG.

ODN	Molecular Weight, Da	−(Δ*f*_s_)_max_, Hz	*K*_D_, nM	*K*_A_, nM^−1^
C-CpG	7115.4	33.9 ± 6.4	59.0 ± 24.0	0.017 ± 0.007
B-CpG	7733.8	26.6 ± 3.8	55.1 ± 17.4	0.018 ± 0.006

## Data Availability

Data is contained within the article or supplementary material.

## Supplementary Materials

The following are available online at https://www.mdpi.com/1422-0067/22/5/2671/s1. Reference [59] are cited in the supplementary materials.
