# Supplementary material for: Magic Peptide: Unique Properties of the LRR11 Peptide in the Activation of Leukotriene Synthesis in Human Neutrophils
Source: Int J Mol Sci. 2021 Mar 6;22(5):2671. doi: 10.3390/ijms22052671 (PMC7961786; doi:10.3390/ijms22052671)

Supplementary

Table S1. Effect of Chloroquine on 5-LOX product formation in PMNLs after ODN priming followed by the addition of opsonized *S. typhimurium* bacteria. Neutrophils (2×10^7) were incubated for 15 min at 37 °C without or with 5 μM Chloroquine, then ODN was added. After 20 min, 0.5×10^9 *S. typhimurium* bacterial cells were added for a further 15 min. Data are presented as mean ± SEM from three independent experiments.

PMNLs+	Σ 5-LOX metabolites (ng/10^7 PMNLs)
Control	22.2 ± 2.2
Chloroquine	19.8 ± 2.7
B-CpG	43.9 ± 4.1
Chloroquine + B-CpG	41.3 ± 4.4
C-CpG	42.1 ± 4.2
Chloroquine + C-CpG	45.2 ± 5.0
A-CpG	18.4 ± 2.1
Chloroquine + A-CpG	21.6 ± 2.5

Figure S1. Neutrophils' viability assessment. PMNLs (10^6 cells/mL HBSS/HEPES) were cultured for 20 min at 37 °C with 5% CO_2 without additives or in the presence of 1 μM ODNs ± 1 μM LRR11. The *S. typhimurium* bacteria were then added for next 15 min. After washing with cold PBS, cells were stained with AnnexinV, conjugated with Alexa Fluor 488, and propidium iodide (PI), followed by flow cytometry analysis (ex. 488 nm, em. 525 nm). Shown are representative dot plots with proportions of viable (region Q1-LL), early apoptotic (region Q1-LR), and late apoptotic and necrotic cells (regions Q1-UR and Q1-UL).

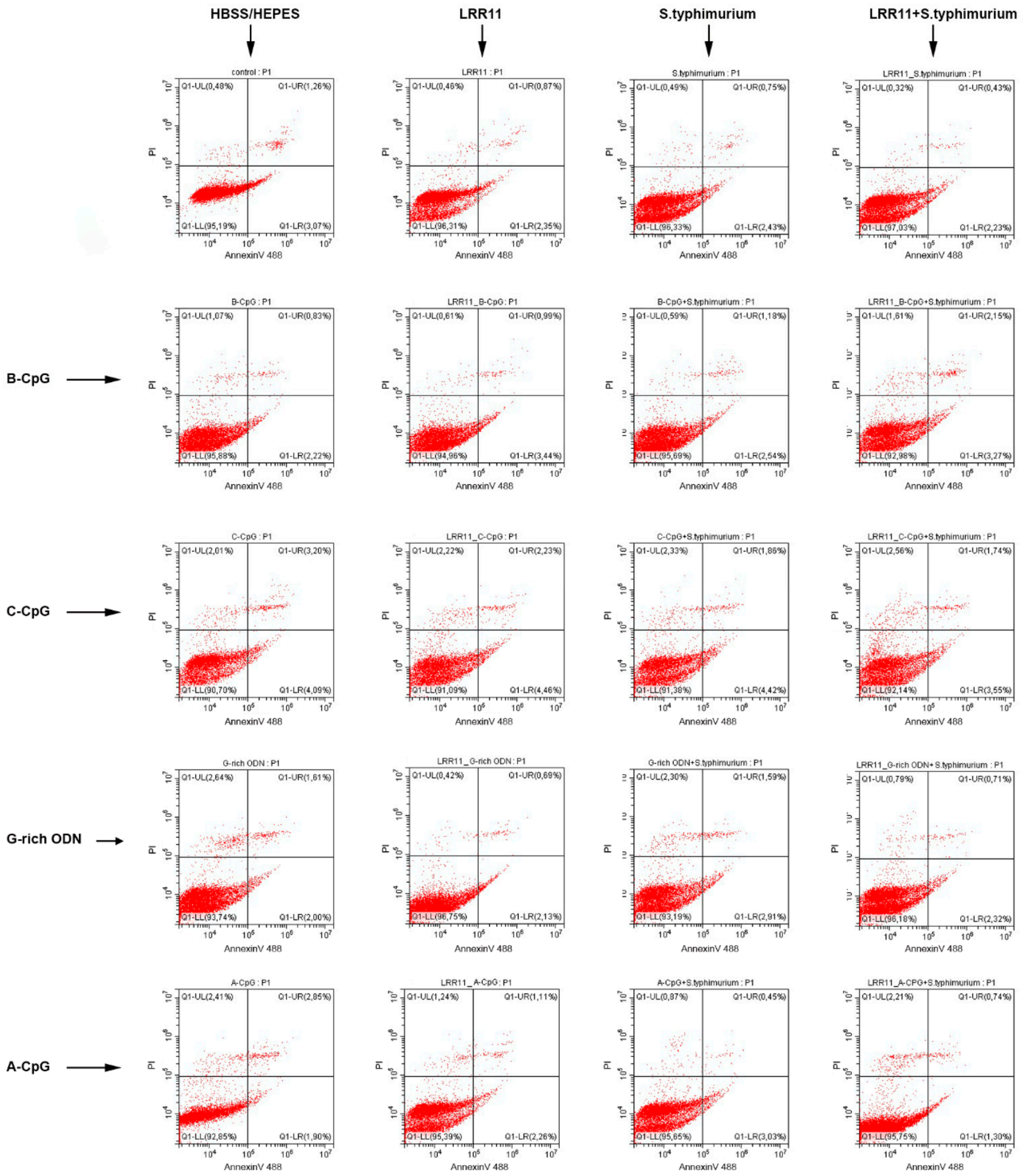

Figure S2a. Effects of ODNs and LRR11 peptide on neutrophil adhesiveness. PMNLs (10^6 cells/probe in HBSS/HEPES) supplemented with 1 μM ODN +/- 1 μM LRR11, except for control samples containing HBSS/HEPES instead of ODNs, were incubated in fibrinogen-coated 24-well plates for 20 min at 37 °C in 5 % CO_2. The supernatants were then carefully removed, and wells were washed twice with warm PBS to remove unattached cells. Adhesion quantitative assessment was carried out as described in [59]. Briefly, hydrogen peroxide (4 mM final concentration) in permeabilizing buffer (67 mM Na_2HPO_4, 35 mM citric acid, 0.1% Triton X-100) supplemented with 5.5 mM ortho-phenylenediamine was added to the attached PMNLs for 5 min. The reaction was stopped by adding of 1M H_2SO_4, followed by recording the absorption values at 492 nm and comparing them with the calibration values. Data are means ± SEM of attached cells amounts, as the percentages of PMNLs initially contained in the samples (n=3; *$p < 0.05$; **$p < 0.01$ in comparison with corresponding controls, as indicated by two-way ANOVA followed by Dunnett's multiple comparison test).

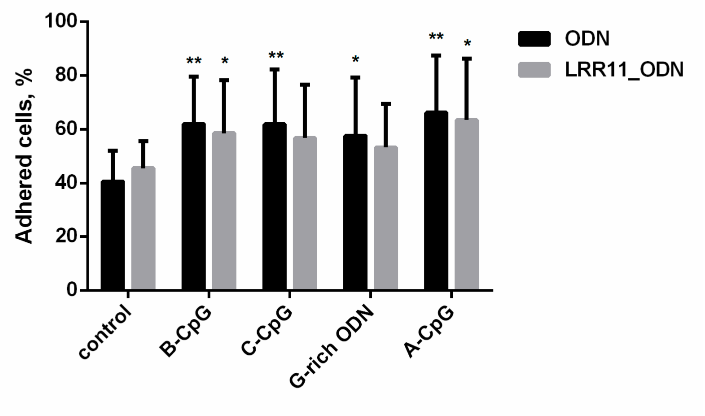

Figure S2b. Influence of LRR11 and ODNs on phagocytic activity of neutrophils. PMNLs supplemented with 1 μM ODN +/- 1 μM LRR11, except for control samples containing HBSS/HEPES instead of ODNs, were incubated for 20 min in Eppendorf tubes at 37 °C in 5% CO_2 with continuous stirring. FITC-labeled *S. typhimurium* were then added for next 20 min, followed by FACS analysis (excitation: 488 nm, emission: 525 nm). Both the relative amounts of PMNLs involved in phagocytosis (**A**, **B**) and the fluorescence intensities, reflecting the average number of bacteria captured per cell (**C**, **D**), were estimated. To distinguish between internalized and surface-bound bacterial cells, trypan blue at a working concentration of 1 mg/ml, was used to quench the surface FITC fluorescence caused by the attached bacteria. Thus, each series of samples was duplicated to obtain results both without (**A**, **C**) and with (**B**, **D**) TB quenching, which corresponds to the total interaction and uptake of bacteria. Averaged flow cytometry data are presented, including means ± SEM for the percentages of FITC-positive (i.e., phagocytic) cells (**A**, **B**), and mean ± SEM for fluorescence intensities of FITC-positive subpopulations (**C**, **D**) (n=3; * $p < 0.05$; ** $p < 0.01$ in comparison with corresponding controls, ns – non significant, as indicated by two-way ANOVA followed by Dunnett's or Tukey's multiple comparison tests).

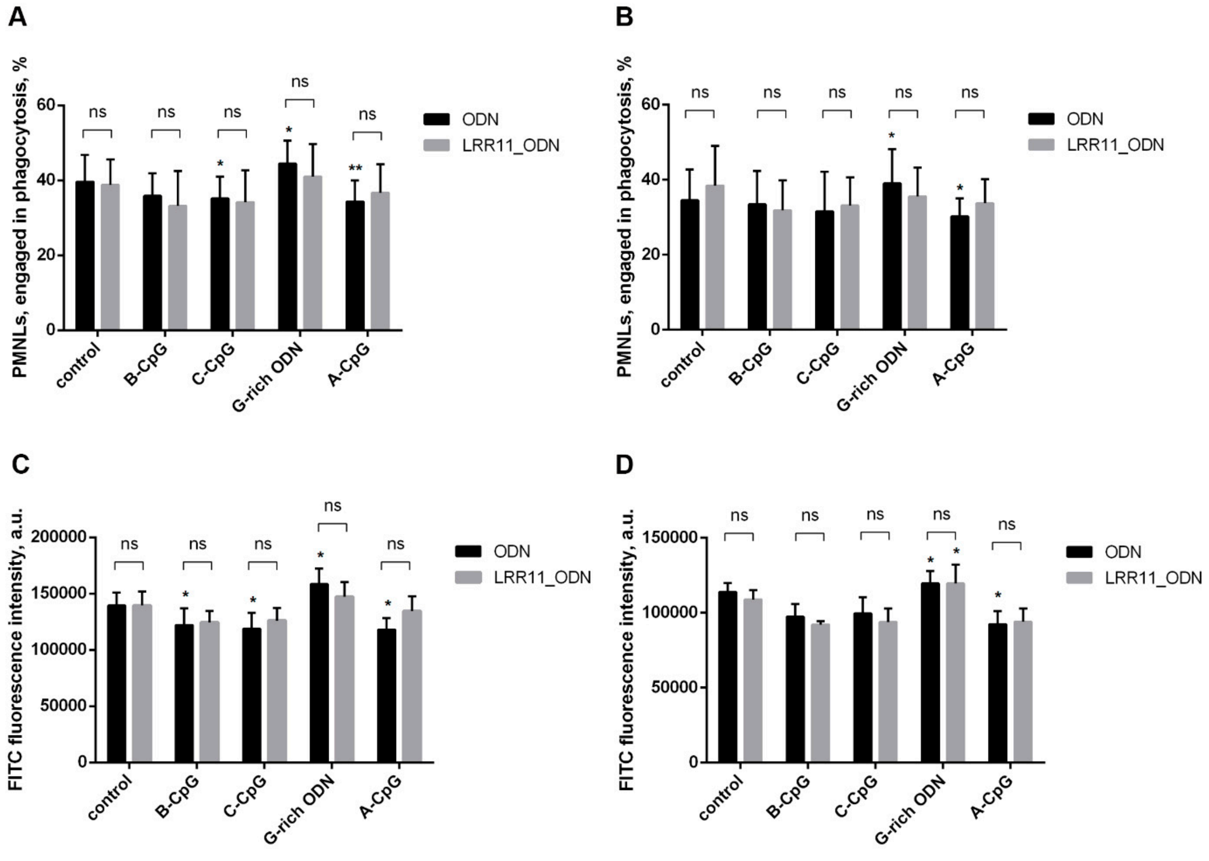

Supplement: Supplementary file 1 [file ijms-22-02671-s001.pdf]
